# Letter II

**Published:** 1804-08-01

**Authors:** 


### LETTER II.

### Of Quacks and Empiricism.

### Character I. Dr. Day.

FROM some fortuitous circumstance, or temporary bias, an ample fortune has been raised by a single nostrum only; and Schomberg's pill, which the late Dr. James procured from a poor German,* and sold under the denomination of James's powder, affords a proof, which the success of a water doctor has fully confirmed. If one specific shall become so productive, it might be concluded that by multiplying the number the product would be proportionally augmented; but to ensure success, it would require an extent of abilities, rarely found in an Empiric: It would be necessary to assume and support different characters, in order to maintain and extend the reputation of each respective specific; but to this versatile exertion Dr. Day proved adequate. He was born in Holland, though of German parents, of the name of Dies, which the Doctor has translated into the English synonism of Day, under which name he first made his appearance in London about 1775, when he kept an elegant equipage with two footmen in green livery; his dress was showy, with brilliant shoe and knee buckles, white silk stockings, and elegantly wrought clothes.

As it was impracticable for Dr. Day, however Proteus-like the various characters he assumed, to appear in each respectively at the same time, he kept Agents, little inferior to himself in artful delusion; Dr. Rock, Dr. Christie, &c. by whom, rendezvous were appointed for the reception of the credulous. Dr. Day himself then lived in Sherrard Street, Golden Square; but at the period I allude to, the name of Rock, who had previously resided with Mayersbach, was upon the door; at the same time he kept a quack warehouse at No. 3, James Street, Covent Garden; Dr. Christie was the bill sticker. Dr. Lantware was another agent, who was appointed to father his famous bill to promote abortion, marked No. 5; but if this did not succeed,

* This occasioned a dispute between him and Dr. James; but the latter was a more expert writer. The former introduced one of his appeals in the public prints with this motto.

"Let Dr. James say what he will,
James's powder is Schomberg's pill."

succeed, a reference was given to the deluded females to consult a lady, who procured accommodations for privately lying-in, as appears by the bill, No. 6.

The originals are in my possession, but I think them so curious and valuable as to merit a place in your Journal.

"To the PUBLIC. (No. 1.)

"The famous *Sympathetic Powders*, which have done so many surprizing cures when all other remedies have failed. As they never can hurt the patient, and are so innocent that young children have been cured by them, the patient has no need of internal medicines, to overload and nauseate the stomach, or hurt the constitution. Every sort of distemper or disease whatsoever, may or will be cured by bringing or sending their morning's urine to the Doctor's house, in Sherrard Street, Golden Square, the third door from Shug Lane, No. 3 over the door, facing a perfumer's.

"The doctor is to be spoke with from nine till two every day, (Sundays excepted). He is also possessed of the most authentic testimonies of the many cures he has performed in the worst disorders. N. B. He cures fits and cancers without fail. Advice to the poor gratis."

"To the PUBLIC. (No. 2.)

"Lately discovered, a new and easy method of curing old standing coughs, consumptions, all disorders of the lungs, asthmatic hooping coughs, tertian and quartan agues, scorbutic and all sorts of eruptions of the skin, cancers, piles, rheumatism, and some other private diseases. Those who are afflicted with any of the aforesaid disorders, may and will be cured by bringing or sending their morning's urine to the Doctor's in Greek Street, Soho, the third door from Compton Street, No. 3 over the door, facing the George. By the doctor's new method, they will relieve in a few days, according to the disorder. He may be spoke with from ten till two every day (Sundays excepted). The doctor is possessed of the most authentic testimonies of the cures he has performed in the most inveterate disorders, and also those that were declared incurable by other gentlemen of the faculty. The Poor advice for nothing.

"N. B. He also cures all disorders of the eyes without fail."

(No. 3.)

"DOCTER CHRISTIE acquaints the public, that he continues disposing of, at the Physical and Mineral Warehouse, in Church Street, St. Ann's, Soho, the second house from from Greek Street, next the Coach and Horses ale-house, and no where else, his anti-venereal treacle, well-known for curing the venereal disease, rheumatism, scurvy, old-standing sores, and breaking out of the skin, at three shillings per bottle, which, by many years experience, has never failed in curing the above mentioned disorders, without hindrance of business, or the knowledge of a bed-fellow; as it works off by urine, it is beyond a doubt superior to any medicine ever invented, in its certainty of performing a safe and easy cure. It is a convenient medicine for Seamen, Travellers, and Servants in places. Those unfortunate people of either sex, who have fallen into the hands of unskilful mechanics, who by getting a medicine call themselves doctors, and give out thousands of puffing bills, and practise on the unhappy people to sell their pernicious drugs, to get money, by keeping them months under a course of physic; let none of those of either sex despair of being cured, though the disease be of long standing.

"Advice gratis — No cure no pay — Genteel people of either sex may lodge and board in the doctor's house."

(No. 4.)

Is by Doctor Christie, and not greatly different from No. 3, but does not offer bed and board for both sexes.

"To the Ladies. (No. 5.)

"The female mixture, which removes all sorts of obstructions in certain cases of ever so old a date, with sore and swelled legs, shortness of breath, giddiness, and all the symptoms which attend a change of life, with ease safety, and certainty.

"*A caution to pregnant women.* As pregnancy is often mistaken for obstructions, whoever has reason to suspect herself with child, must not use this powerful mixture, as *it will certainly bring on a miscarriage.*

"It is sold at the Physical and Mineral Warehouse, in Church Street, St. Ann's Soho, the second house from Greek Street, next door to the Coach and Horses ale-house, and no where else, at half a guinea, and a guinea a bottle, with proper directions how to use it."

(No. 6.)

Is advertised "A Gentlewoman," who sells a mixture for 3 shillings a bottle to remove weaknesses, and concludes thus. "To the Ladies.

"N. B. Pregnant women may be accommodated according to their abilities, *where privacy is required.* No. 3 James Street, Covent garden."

One

One might have imagined, that in a well regulated city, the magistrates would not have suffered the quack bill, No. 5, to have been circulated with impunity; as infanticide has been amenable to law in every civilized nation. Whoever contemplates the Institution of the Royal College of Physicians, would have concluded that its salutary interference would have been laudably exerted upon such a gross insult to virtue, and open violation of law; but fortunately for Dr. Day, he was not a licentiate, but merely a German adventurer; and it has been principally against the former, that its vengeance has been exercised.

It ought, however, to be recorded, in extenuation of Dr. Day, that his medicine to promote abortion, was an harmless deception, although it was found that the demand for it was so great, as to have enabled him to pocket as much as ten guines a day; till the deluded females found it inactive, and consequently not injurious to their health; and perhaps, by adopting this means of deluding them, he saved them from attempting some really dangerous expedient; for I believe that Dr. Day, as it has been observed, never administered any pernicious means for the purpose of inducing abortion, however censurable he might have been, in imposing upon the weakness of the public; he was naturally good tempered, and facetious, which led him to laugh at, rather than correct, their credulity; and he might certainly be considered as very officious and gallant, in gratifying the wishes of the female sex, in not only offering them a bed as well as the other sex, but likewise a remedy, to rid them of an unwelcome burthen; and in case of its failure, affording them private apartments for their easy accommodation. It was, hence, doubly his interest, to prevent infanticide; for in addition to his abortive mixture, he had the profits of an accoucheur, and the hire of chambers suitably to the rank and finances of the victim of double deception, to avail himself of.

I have not seen the doctor for some years past, but I am informed that he resides at the house from whence one of his quack bills is dated, and that he claims the respect of many of his neighbours, as a private individual which induces me to think that he merits a place in your Journal, prior to Dr. Griffenbergh and Dr. Mayersbach, who will be introduced in the next letter of

Your Constant Reader,

*London, June* 30, 1804.

IETROS.

