# Corrigenda

**Published:** 1804-09-01

**Authors:** 


### CORRIGENDA.

Page 137, line 6, after the last word, *James*, add, *it is thought*.
137, for *Dr. Rock*, read *Dr. Koch*.

